# Supplementary material for: Separable cognitive and motor decline tracked across adult life-span for goal-directed navigation
Source: iScience. 2025 Aug 26;28(10):113441. doi: 10.1016/j.isci.2025.113441 (PMC12481093; doi:10.1016/j.isci.2025.113441)
Supplement: Document S1. Figures S1–S8 and Table S1 [file mmc1.pdf]

iScience, Volume *28*

Supplemental information

Separable cognitive and motor decline tracked across adult life-span for goal-directed navigation

Gian Luca Lancia, Marco D'Alessandro, Mattia Eluchans, Miguel Ibáñez-Berganza, Hugo J. Spiers, and Giovanni Pezzulo

Supplementary materials

Supplementary figures

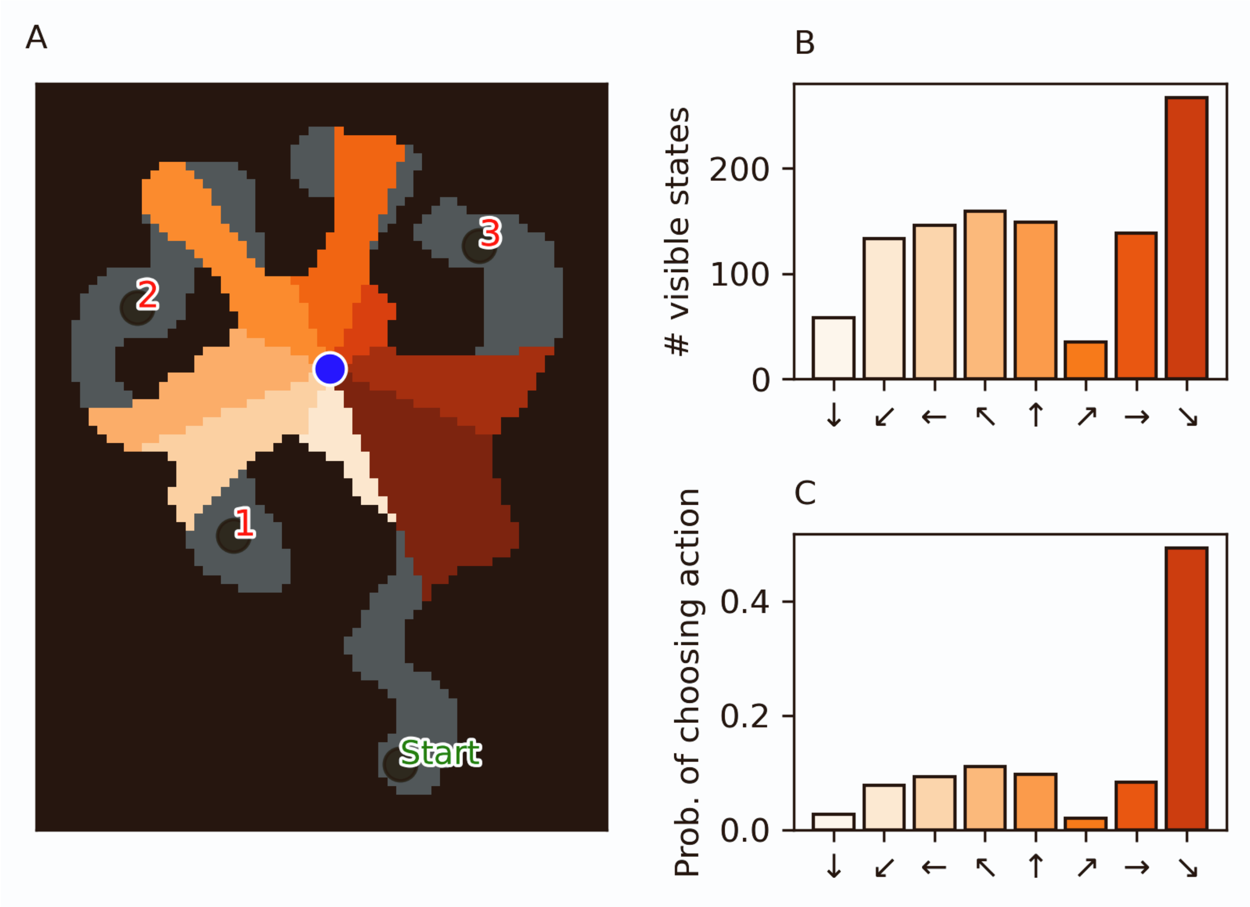

Figure S1: An example illustrating the Visibility-based model. Related to Figure 2 and Figure 3. A) Area of the map visible from the point of view of a simulated player (blue circle) in a given position, of level 46 from the center of the map. The visible area is subdivided in 8 bins, each spanning an angle of 45°. Grey areas are not visible. B) Histogram showing the number of states in each cone. C) An example of softmax function applied to the number of states visible in a bin to obtain the probability of choosing the related action. See main text for explanation.

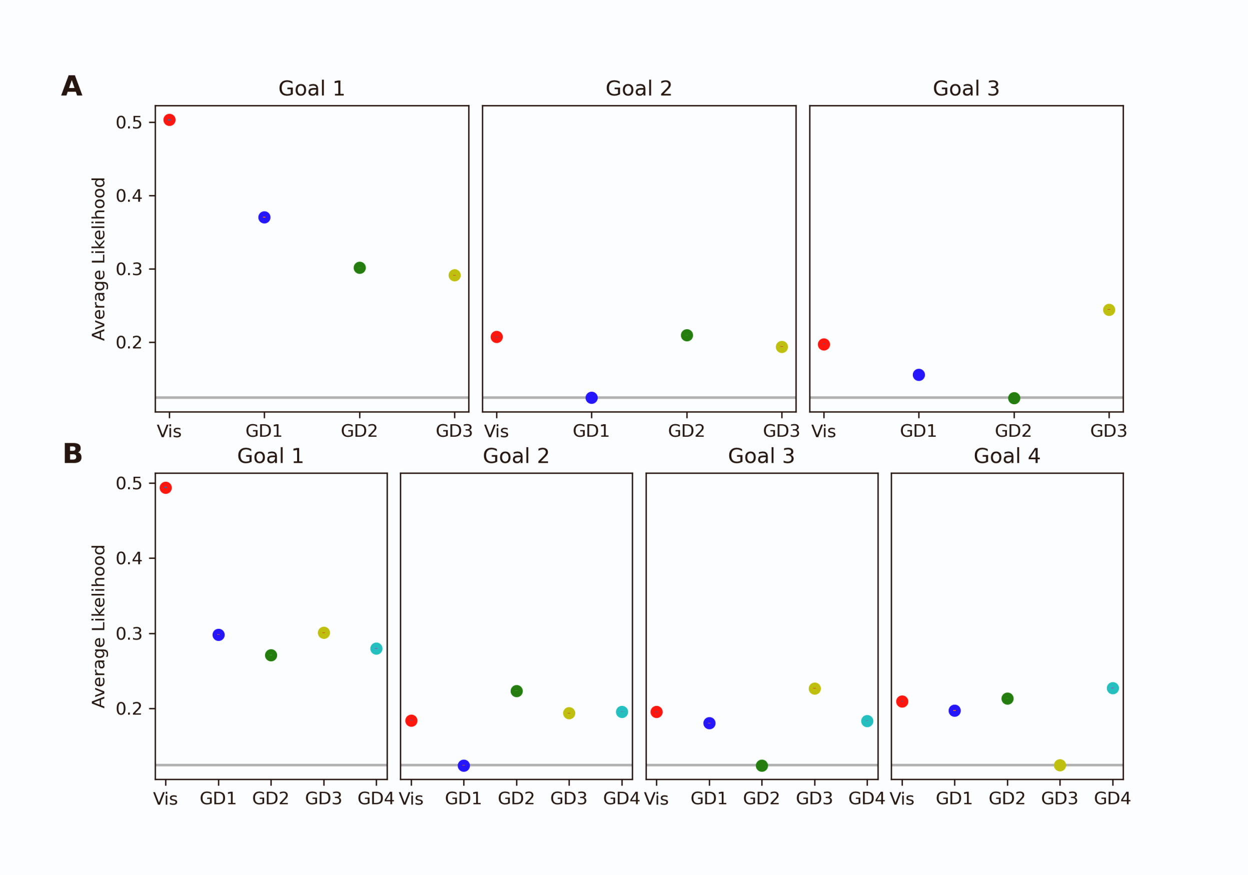

Figure S2: Model Performance by Goal and Level but for the first 10 steps. Related to Figure 5. Averaged likelihoods of the models over participants, levels, and trajectory segments. Error bars represent standard errors, which are smaller than the depicted symbols. The models are color-coded: red for Visibility, blue for Goal-Directed to Goal 1, green for Goal 2, yellow for Goal 3, and azure for Goal 4. Panel A (top row) displays model likelihoods for levels with three goals, with subplots for each goal reflecting model performance as participants navigate towards each successive goal. Panel B (middle row) shows likelihoods for levels with four goals

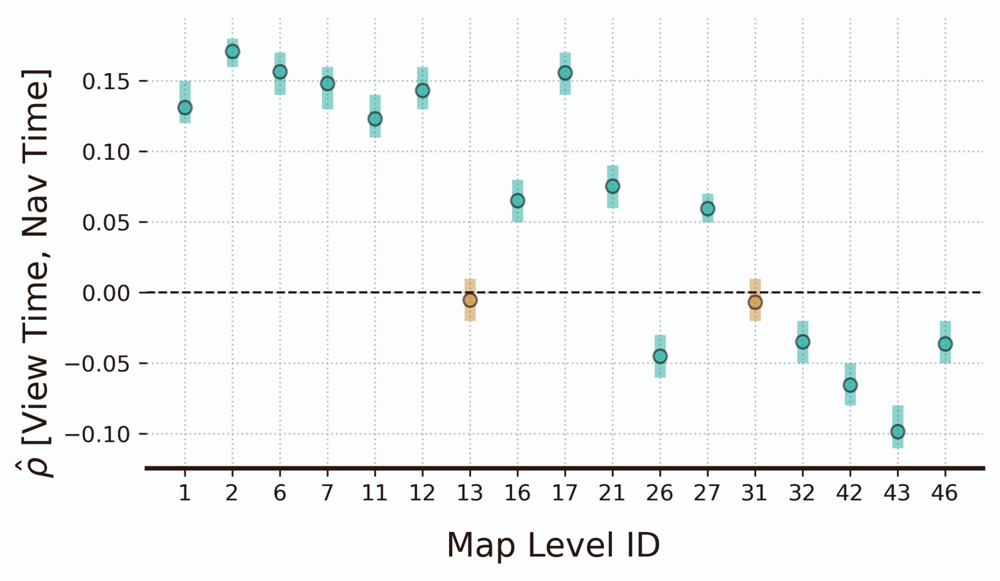

Figure S3: Within-map correlation analysis of map navigation time and map view time. Related to Figure 7 and Figure 2. Correlation values between navigation time and view time are depicted for every map level. Dots represent the correlation effect size with 95% confidence intervals error bars. Green and brown colors represent significant and non-significant correlation values, respectively. Our results show that, for 10 out of 17 levels, there is a significant positive correlation between view time and navigation time, indicating that participants who spent more time viewing the level tended to navigate it more slowly. For 5 levels, we observed a significant negative correlation, suggesting that longer viewing was associated with faster navigation. Notably, 4 of these 5 levels contain 4 goals, which may indicate a more demanding planning process (the only other level with 4 goals not included in this group is level 27). Finally, for 2 levels, the correlation between view time and navigation time was not significant.

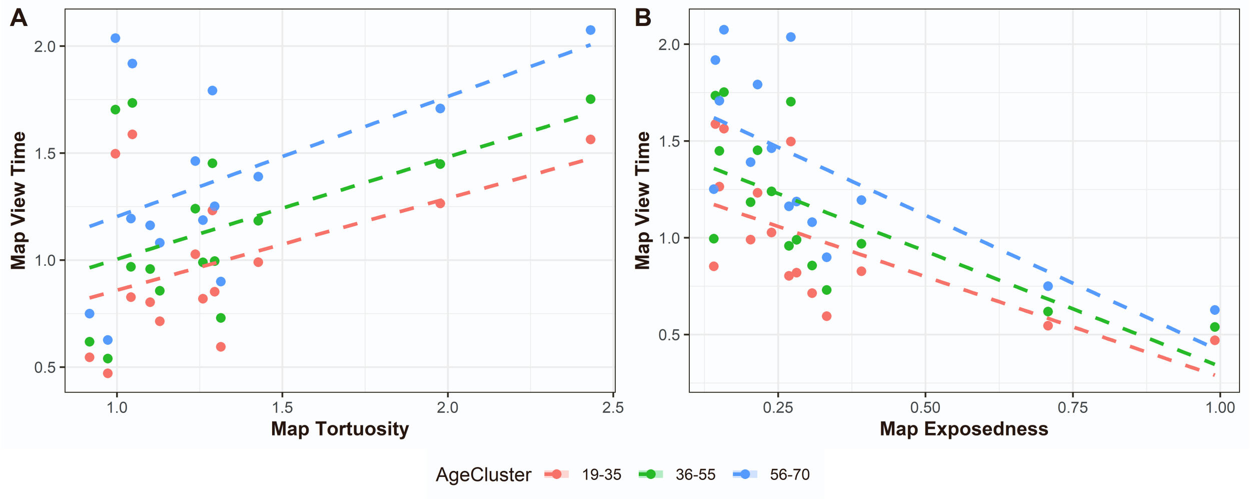

Figure S4: Relationship Between Map View Time and Map Characteristics Across Age Clusters. Related to Figure 7 . We performed two separate mixed-effect regression analyses with subjects as random effects. The map's view time is used as the dependent variable in the two map-level analyses, and Map Tortuosity and Map Exposedness as predictors, respectively for each analysis. For all the analyses, clustered Age (19-35, 36-55, 56-70) was used as a covariate. Each dot represents the value averaged across all subjects in a given level, color-coded by age cluster (red for 19-35, green for 36-55, and blue for 56-70). The dashed lines represent the linear regression fits for each age cluster, indicating an increase in map view time with increased Map Tortuosity. Results of the first analysis show a significant increase in the map's view time as Map Tortuosity increases ($\beta = 0.39$, $t(1.7 \times 10^5) = 90.78$, $p < 0.0001$), and a significant effect of age. A) Scatter plot showing the relationship between map view time and Map Tortuosity. Furthermore, a contrast analysis revealed that intercepts increase for older age clusters, such that the overall map's view times is higher for the 36-55 age cluster compared to 19-35 ($\beta = 0.158$, $z = 10.97$, $p < 0.0001$), and higher for 56-70 compared to 36-55 ($\beta = 0.22$, $z = 14.31$, $p < 0.0001$). B) Scatter plot illustrating the relationship between map view time and Map Exposedness. Each dot represents the value averaged across all subjects in a given level, color-coded by age cluster (red for 19-35, green for 36-55, and blue for 56-70). The dashed lines show the linear regression fits for each age cluster, indicating a decrease in map view time with increased Map Exposedness. Results of the second analysis show a significant decrease in the map's view time as Map Exposedness increases ($\beta = -1.48$, $t(1.7 \times 10^5) = -147.29$, $p < 0.0001$), and a significant effect of age on the intercepts as well. In particular, the overall map's view times is higher for the 36-55 age cluster compared to 19-35 ($\beta = 0.16$, $z = 11.05$, $p < 0.0001$), and higher for 56-70 compared to 36-55 ($\beta = 0.23$, $z = 14.53$, $p < 0.0001$). Altogether, these findings indicate that individuals require more time to encode maps that exhibit greater difficulty (characterized by increased tortuosity) and those that prospect more challenging navigation (indicated by lower exposedness), as well as that younger individuals are faster at encoding maps overall. We also tested whether there is a possible correlation between view time and goal-directedness, but the correlation does not reach significance ($p > 0.5$).

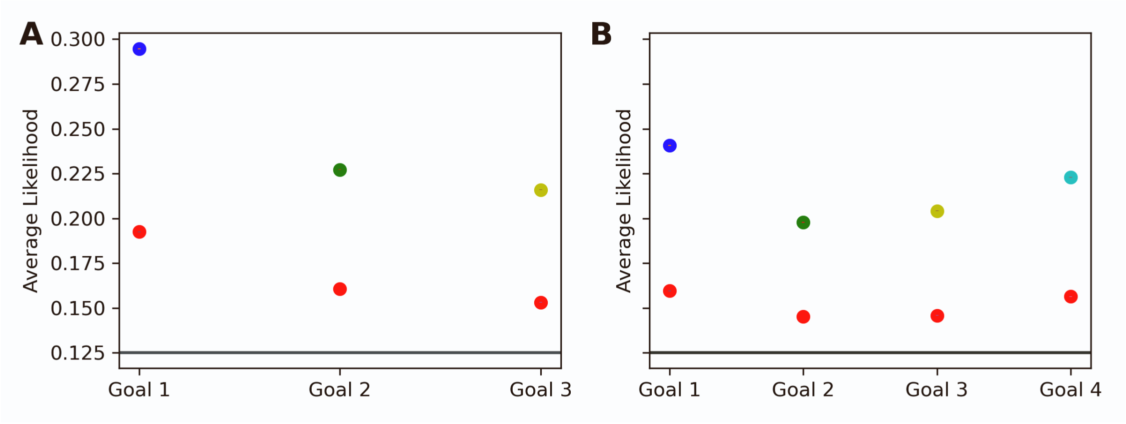

Figure S5: Likelihood of the relevant Goal-Directed (GD) models. Related to Figure 5. Panels A and B summarize the likelihood of the relevant Goal-Directed (GD) models for levels with three and four goals, respectively, plotting only the likelihoods for the ”correct” GD model for each goal (blue, green, yellow, azure) alongside the Visibility-based model (red).

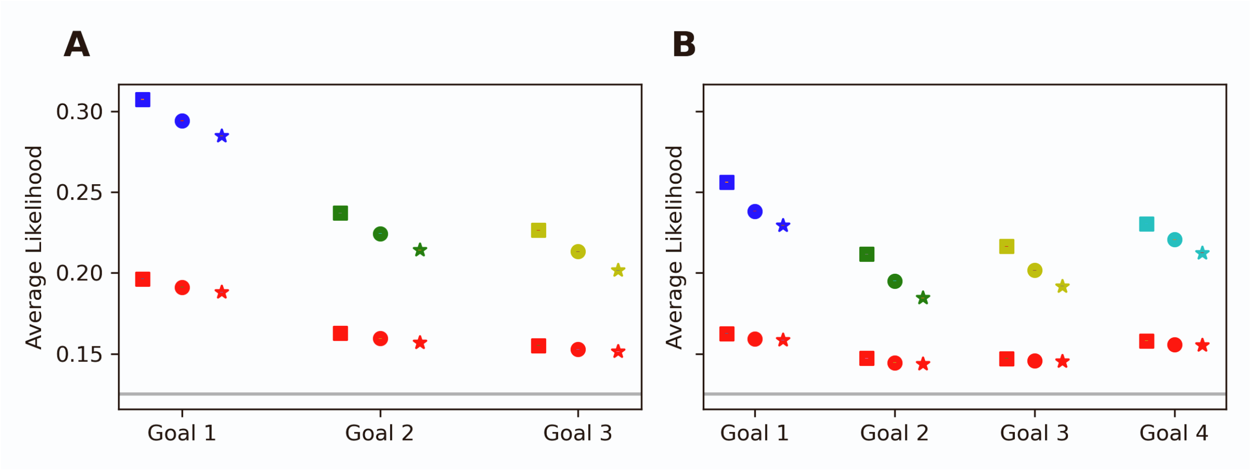

Figure S6: Model Performance by Goal, Level and Age Cohort. Related to Figure 5. Averaged likelihoods as in Figure S5, but grouped by age: square markers for participants aged 19-35, circle markers for participants aged 35-55, and triangle markers for participants aged 55-70. The likelihoods are calculated in the same manner as previously described, with trajectory segments defined by goal reaching within each level. The models remain color-coded for ease of reference: Visibility (red), Goal-Directed model to Goal 1 (blue), Goal 2 (green), Goal 3 (yellow), and Goal 4 (azure).

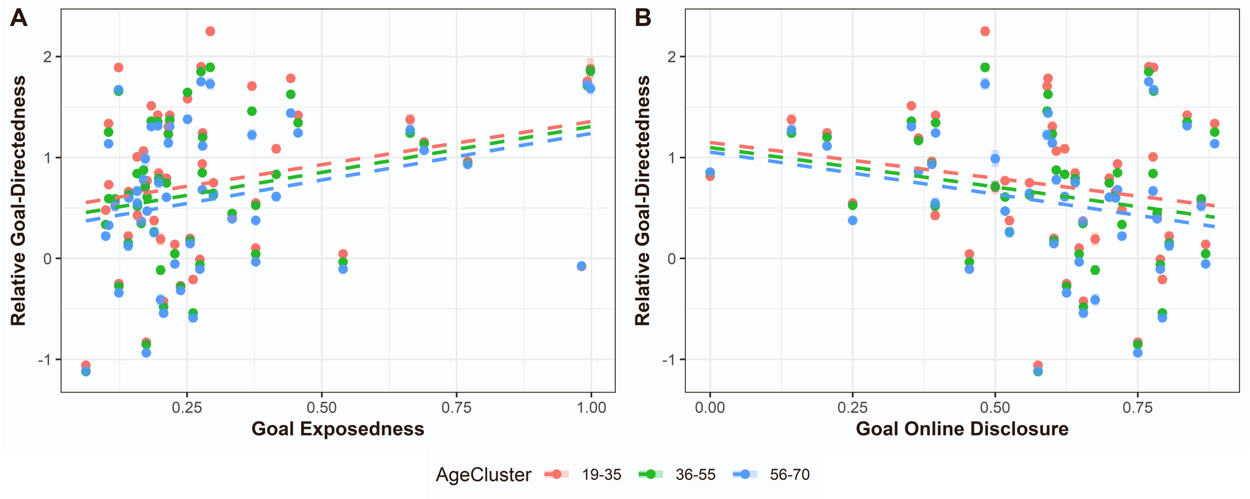

Figure S7: Influence of Goal Exposedness and Goal Online Disclosure on Relative Goal-Directedness Across Age Clusters. Related to Figure 9. We performed a mixed-effect regression analyses with subjects as random effects. For all the analyses, the age of participants clustered in three cohorts (19-35, 36-55, 56-70) was used as a covariate. Relative Goal-Directedness was first estimated given the model in eq. 6 for each subject and each goal, and its logit was used as the dependent variable. Goal Exposedness and Goal Online Disclosure were used separately as predictors for each analysis, respectively. A) Scatter plot illustrating the relationship between Relative Goal-Directedness and Goal Exposedness. Each dot represents a level, color-coded by age cluster. The dashed lines represent the linear regression fits for each age cluster, showing a positive trend between Relative Goal-Directedness and Goal Exposedness across all age groups. Results of the first analysis show a significant increase in Relative Goal-Directedness as Goal Exposedness increases ($\beta = 1.12$, $t(1.7 \times 10^5) = 65.42$, $p < 0.0001$) and a significant effect of age such that the overall Relative Goal-Directedness is higher for the 19-35 age cluster compared to 36-55 ($\beta = 0.05$, $z = 5.85$, $p < 0.0001$), which in turn shows higher values compared to 56-70 ($\beta = 0.06$, $z = 6.43$, $p < 0.0001$). B) Scatter plot depicting the relationship between Relative Goal-Directedness and Goal Online Disclosure. Each dot represents a level, color-coded by age cluster. The dashed lines show the linear regression fits for each age cluster, indicating a slight decrease in Relative Goal-Directedness with increasing goal online disclosure. The data suggests that age clusters exhibit similar trends in Relative Goal-Directedness relative to goal characteristics. Results of the second analysis reveal a significant decrease in Relative Goal-Directedness as Goal Online Disclosure increases ($\beta = 0.62$, $t(1.7 \times 10^5) = -45.61$, $p < 0.0001$) and a significant effect of age on the intercept. Even in this case, the overall Relative Goal-Directedness is higher for the 19-35 age cluster compared to 36-55 ($\beta = 0.05$, $z = 5.55$, $p < 0.0001$), which in turn shows higher values compared to 56-70 ($\beta = 0.057$, $z = 5.82$, $p < 0.0001$).

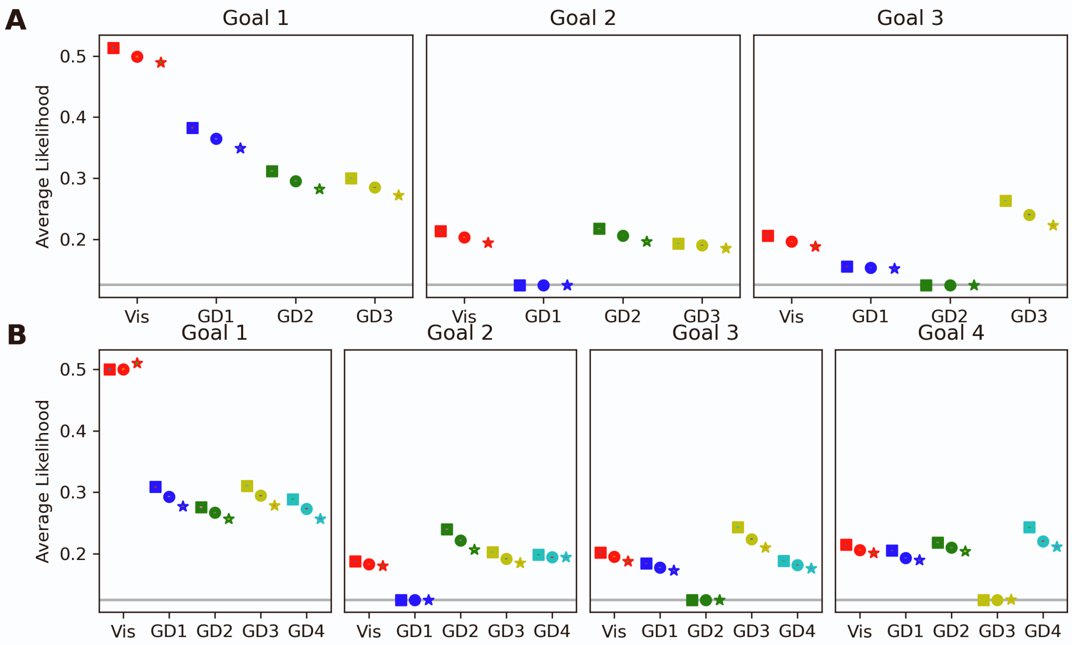

Figure S8: Model Performance by Goal, Level and Age Cohort but for the first 10 steps. Related to Figure 11. Averaged likelihoods grouped by age: square markers for participants aged 19-35, circle markers for participants aged 35-55, and star markers for participants aged 55-70. The likelihoods are calculated in the same manner as previously described, with trajectory segments defined by goal reaching within each level. The models remain color-coded for ease of reference: Visibility (red), Goal-Directed model to Goal 1 (blue), Goal 2 (green), Goal 3 (yellow), and Goal 4 (azure

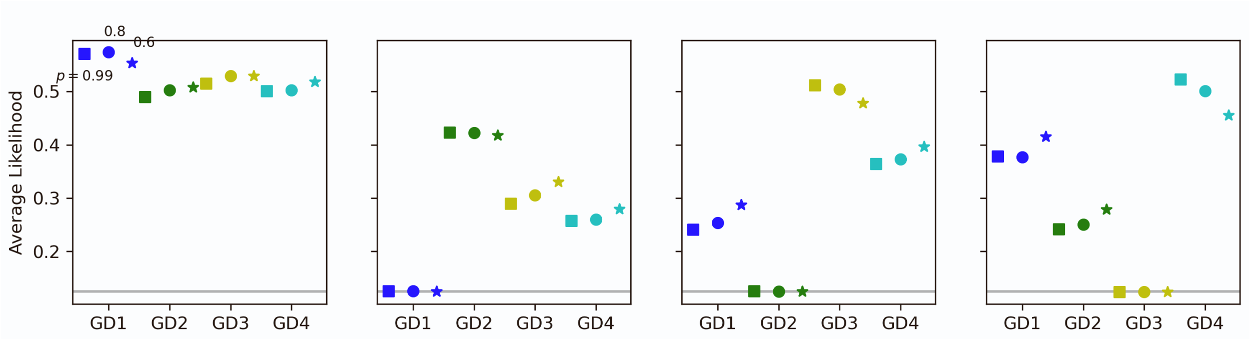

Figure S9: Model Performance by Goal, Level, and Age Cohort in Early Trajectory Phases with data simulated using a Sequencing Error model. Related to Figure 11. The figure shows the averages of likelihoods of the Sequencing Error model's simulated trajectories evaluated at three distinct values of p ($p = \{0.99, 0.8, 0.6\}$). For simplicity, simulation was performed for the 10 time steps of each problem, which are the most discriminative. See the main text for explanation.

Supplementary tables

Table S1: ANOVA Summary Results. Related to Figure 5.

N_{goals}	Goal ID	F_{value}	DoF	p_{value}
3	1	92097.0684	3	$\ll 10^{-3}$
3	2	167567.9079	3	$\ll 10^{-3}$
3	3	219396.6102	3	$\ll 10^{-3}$
4	1	81737.4851	4	$\ll 10^{-3}$
4	2	72731.2548	4	$\ll 10^{-3}$
4	3	52497.7072	4	$\ll 10^{-3}$
4	4	74606.1757	4	$\ll 10^{-3}$
